# Haemorrhoids and Their Treatment

**Published:** 1881-04

**Authors:** A. Charge

**Affiliations:** Paris; France


### HÆMORRHOIDS AND THEIR TREATMENT.

BY DR. A. CHARGE, FRANCE.

[Translated and condensed from "La Biblioteque Homœopathique," Feb., 1881.]

THE principal symptoms of hæmorrhoidal disease are: a constant congestion at lower portion of rectum; tumors, formed by a varicose dilatation of the veins at lower extremity of rectum and at anus; first a discharge of blood, and later, a whitish mucoid discharge.

SYMPTOMS OF HÆMORRHOIDAL INFLAMMATION.—Heaviness of the head, cephalalgia, vertigo; digestive troubles, such as loss of appetite, eructation; sensation of fullness in the abdomen with pressure in rectum and at anus. Patient is gloomy, despondent and very irritable; is not comfortable in any position, either sitting or standing. Pulse is full and hard. Constipation, with frequent ineffectual desire for stool. There is heat, itching and pricking, at anus; heat in the urethra and bladder during micturition. Defecation is difficult, painful and often accompanied by a slight discharge of blood. In slight cases, this discharge of blood terminates the inflammatory trouble.

SYMPTOMS OF HÆMORRHOIDAL TUMORS.—These may be internal or external; flowing or dry (blind). The internal are hid in the rectum and only protrude from the anus when straining at stool. These internal tumors may be protruded by persistent straining or by increased congestion; they are then either reducible or irreducible tumors. The reducible tumors are of minor importance; while the irreducible are most troublesome and are accompanied by inflammation, heat, and tenesmus of bladder and rectum. The tumors are of purplish hue at first, then they become darker, finally even black and gangrenous, caused, perhaps, by strangulation. The hæmorrhoidal blood is nearly always black, and escapes most frequently at stool. This hemorrhage is caused by either a rupture or an engorgement of the hæmorrhoidal veins. The blood lost is variable in amount and frequency of occurrence. Following the bloody discharge there may appear a whitish mucoid discharge, arising from the continuance of the inflammatory trouble, which in its turn is caused by some particular dyscrasia.

CAUSES.—Constipation is often cited as the cause of hæmorrhoids. This is false in theory; constipation is certainly a complication of hæmorrhoidal disease, but there is no valid relation between them, as of cause and effect. Hæmorrhoids are very often seen, it is true; but constipation is still more prevalent.

[We might put it thus: All hæmorrhoidal patients have not constipation; nor have all constipated persons hæmorrhoids; the case is merely this, most hæmorrhoidal persons are constipated, and the *cause* of one must be the cause of *both*.—ED.]

The true cause of hæmorrhoids is an internal condition caused by a miasmatic infection of the system, nearly always of a psoric nature. I wish to give here another proof of the truth of the Hippocratean aphorism, so often seen in these cases—*Naturam morborum ostendit curatio.* The good effects of *Sulphur* upon these hæmorrhoids, so real, so constant, serves to show the psoric origin of this disease.

Hæmorrhoids being caused by an internal, general and constitutional trouble, for that very reason the flow should not be suddenly checked, nor the tumors treated by improper applications nor by revulsive medicines, nor by knife or cautery. The knife cuts, the cautery burns, but neither reaches the real cause of the disease. The radical cure of any malady, proceeding from an internal cause, can only be obtained by attacking the primary cause of the disease; so in hæmorrhoids the flow and the tumors are lesions, which if simply suppressed without curing their cause, what happens most frequently? New lesions, graver, perhaps, will follow, and endanger the life of the patient. We see every day, as a consequence of such brutal suppression of hæmorrhoids, cerebral congestion, apoplexy, softening of the brain, incurable dyspepsias, etc.

The physician, then, avoiding dangerous palliation, should always consider the necessity of remembering the general disease which causes the hæmorrhoids, and so painful and so various are the sufferings of the hæmorrhoidal patient, that he should treat them only by specific remedies; that is, by medicines whose pathogenesis shows them to be especially fitted to the individual case under observation. For it is the homœopathicity of remedies which proves their specificity and gives the reason for it.

The old school is nowhere poorer than in the treatment of hæmorrhoids; *we* do not make this charge; it is sufficient for us not to deny the truth of their own confessions.

"The treatment of hæmorrhoids is, without contradiction, one of the richest" (rich in words! for pathology without therapeutics can not be considered as rich as medicine which claims to be a healing art), "as no part of medicine has been more diligently studied. Still, it is a confused mass of varying methods, through which one must search diligently to find those having practical experience in their favor." ("Valleix," vol. iii. p. 89).

Is this clear? poor young physicians! what can you gather from it? And you are not yet at the end of your troubles; for they tell you of hot baths, of cold baths, of cold lotions, of astringents, narcotics, purgatives, etc. Choose your method, if you dare.

"We are little in favor of prolonged baths, having observed that their use prolonged the trouble and increased the inflammation." (*Dict. de med. et de chir. pratique*, vol. xvii, p. 425). Cold lotions and cold seat [or hip] baths. "All physicians consider these means as very dangerous." "Astringents act in same manner as cold water and are also regarded as very dangerous." (Valleix, eod. loc.). "Narcotics fail completely to do any good, purgatives render the constipation more persistent," etc.

Enough, enough! Yet we were informed at the commencement, that the treatment of hæmorrhoids was, without contradiction, one of the richest! Judge, then, of the rest.

Surgery avenges the incapacity of the physician, but at what a price!—ligatures, excision, the cautery, caustics, liquid and solid—the cost is paid by the patient!

No, we can gather nothing useful from the old school treatment of hæmorrhoids; but yet, we are glad to be able to commend their hygienic and dietetic regulations. The patient should avoid spices, etc., alcoholic drinks, strong wines, tea and coffee. Digestion can be aided by daily out-door exercise and by using ripe fruits, fresh vegetables, etc.

TREATMENT.

Hæmorrhoids, painful: *Ant. crud.*, *Ars.*, *Bell.*, *Ignat.*, *Lycop.*, *Puls.*, *Sed. teleph.*

H. with nightly aggravation: *Ars.*, *Collins.*, *Carbo veg.*

— — evening aggravation: *Alum.*, *Am. m.*, *Collins. Platina*, *Pulsat.*

— — morning aggravation: *Nux vom.*, *Podoph.*

— — amelioration from the night's rest: *Alumina.*

— — amelioration from cold water: *Aloes*, *Apis.*

— dry, blind: *Aesculus hipp.*, *Collins.*, *Graph.*, *Sed. teleph.*, *Sulph.*

— flowing, bleeding: *Acon.*, *Am. c.*, *Bell.*, *Caps.*, *Erigeron*, *Ferr.*, *Hamamelis*, *Ign.*, *Ipec.*, *Millefol.*, *Kreos.*, *Nux vom.*, *Platina*, *Trilli pend.*

— with mucous discharge: *Ant. crud.*, *Carbo veg.*, *Colchic.*, *Merc.*, *Puls.*

— — muco-sanguinolent discharge: *Ant. cr.*, *Borax*, *Ign.*, *Merc.*, *Puls.*

— — muco-purulent discharge: *Ant. cr.*, *Hepar*, *Lyc.*

— protruding: *Aloes*, *Amm. c.*, *Ars.*, *Carbo veg.*, *Graphites*, *Mur. ac.*, *Nitric acid.*

— easily replaced: *Ignatia.*

— irreducible: *Atropia sulph.*, *Ars.*, *Bell.*, *Silicea.*

— with colic: *Ant. cr.*, *Coloc.*, *Nux vom.*, *Puls.*

— — flatulence: *Ant. cr.*, *Ignat.*, *Lyco.*, *Phos.*, *Ph. acid.*

— — pain in lumbar regions: *Bell.*, *Nux vom.*, *Rhus t.*

— — tenesmus: *Aloes*, *Merc.*, *Platina*, *Ph. acid.*

H. with constipation: *Ant. crud.*, *Alumina*, *Aeculus hipp.*, *Collins.*, *Lyco.*, *Nux vom.*, *Sulph.*

— — alternation of constipation and diarrhœa: *Ant. crud.*

— — diarrhœa: *Aloes*, *Capsic.*, *Podophyl.*

— — falling of rectum: *Ignat.*, *Graph.*, *Lyc.*, *Natr. mur.*, *Nux vom.*, *Podoph.*, *Sulph.*

— — falling of rectum while urinating: *Muriatic Acid.*

— — eczema of perineum: *Hepar*, *Petroleum.*

— — herpetic eruptions around anus: *Hepar*, *Lyco.*, *Merc.*, *Natr. mur.*, *Petrol.*, *Sepia.*

— — pustulus eruption: *Amm. mur.*, *Caustic.*, *Kali carb.*

— — fistula: *Hydrastis*, *Silicea*, *Sulphur.*

— — fissures: *Arsenic.*, *Caust.*, *Graph.*, *Nitr. ac.*, *Ledum teleph.*, *Petrol.*, *Ratanhia.*

— — ulceration: *Phosphorus.*

— — dysuria: *Belladonna.*

— in infants: *Amm. c.*, *Borax*, *Collins.*, *Merc.*

— — old people: *Amm. c.*, *Anacardium.*

— during pregnancy: *Nux vom.*, *Lycopodium.*

— — confinement: *Pulsatilla.*

— in clerks: *Nux vomica.*

— — drunkards: *Ars.*, *Nux vom.*, *Lachesis.*

(To be continued).

